# RISUG^®^ as a male contraceptive: journey from bench to bedside

**DOI:** 10.1186/s12610-020-0099-1

**Published:** 2020-02-13

**Authors:** Barkha Khilwani, Ayesha Badar, Abdul S. Ansari, Nirmal K. Lohiya

**Affiliations:** Centre for Advanced Studies, Department of Zoology, University of Rajasthan, Jaipur, 302004 India

**Keywords:** Male contraception, RISUG^®^, Clinical trials, Azoospermia, Reversibility, Contraception masculine, RISUG^®^, Essais cliniques, Azoospermie, Réversibilité

## Abstract

Even after decades of research men still lack reliable and reversible contraceptive methods comparable to female methods of contraception. Traditional methods of male contraception present a high failure rate and also involve high risk both when used for contraception and for protection against sexually transmitted diseases. Various chemical, hormonal, immunological, vas based and herbal methods of contraception have been examined by scientists world over during the past four decades. Among the possible lead approaches, exogenous hormonal contraception, either alone or in combination with progesterone or antiandrogen, is being viewed at low profile because of their insufficiency in inducing uniform suppression of spermatogenesis and steroid related long term complications. As an alternative to vasectomy, among various intravasal devices being examined, RISUG^®^ (Reversible Inhibition of Sperm Under Guidance), a co-polymer of styrene and maleic anhydride offers long term contraception with safety, efficacy and it can be delivered by no-scalpel injection. Thus it is the only male contraceptive procedure currently under Phase- III Clinical Trial. The non-invasive reversal technique, successfully demonstrated in langur monkeys and functional reversal achieved with dimethyl sulphoxide (DMSO) and sodium bicarbonate (NaHCO_3_) in rats and rabbits with safety at F_1_ generation (first filial generation) have projected RISUG^®^ as a better alternative to vasectomy. In this narrative review we revisit the long journey of RISUG^®^ beginning with formulation on a bench towards reaching the market as a safe and effective contraceptive method, discussing various milestones and roadblocks of this expedition awaiting the mandatory regulatory clearance from the Government of India. Successful completion of ongoing phase III clinical trials with demonstration of reversal in human volunteers will give an indigenously developed male contraceptive to the world.

## Résumé

Malgré plusieurs décennies de recherche, il manque toujours pour les hommes des méthodes de contraception fiables et réversibles qui soient comparables aux méthodes de contraception féminine. Les méthodes de contraception masculine traditionnelles ont un taux d’échec élevé ; elles sont aussi à risque lors d’utilisations à la fois comme contraceptif et comme protection contre les infections sexuellement transmissibles. Au cours des 40 dernières années, le monde scientifique a évalué différentes méthodes de contraception masculine basées sur des approches chimiques, hormonales, immunologiques, déférentielles ou à base de plante.

Parmi les pistes possibles d’approche, la contraception par apport d’hormone exogène, soit seule soit associée à un progestatif ou à un anti androgène, est actuellement perçue comme ayant un faible profil en raison de son incapacité à induire une suppression uniforme de la spermatogenèse et des complications à long termes des stéroïdes.

Parmi les alternatives à la vasectomie, plusieurs dispositifs intra déférentiels ont été évalués dont le RISUG^®^ (Reversible Inhibition of Sperm Under Guidance). Ce dernier est un copolymère de styrène et d’anhydride maléique qui offre une contraception de longue durée avec innocuité et efficacité, pouvant être mise en place par injection sans scalpel. C’est ainsi actuellement la seule procédure contraceptive masculine pour laquelle un essai clinique de Phase-III est en cours. La technique de réversibilité sans scalpel, démontrée avec succès chez le singe *Langur*, ainsi que la réversibilité fonctionnelle par le sulfoxyde de diméthyle (DMSO) et le bicarbonate de sodium (NaHCO_3_) confirmée chez le rat et le lapin, sans atteintes sur la génération F1 (première génération de petits), ont fait apparaître le RISUG^®^ comme une meilleure alternative à la vasectomie.

Dans cette revue narrative, nous réévaluons le long chemin du RISUG^®^ depuis une formulation de paillasse de laboratoire jusqu’à sa mise sur le marché comme méthode de contraception sans risque et efficace, en discutant les différents jalons et obstacles rencontrés au cours de cette expédition dans l’attente de l’autorisation réglementaire obligatoire du Gouvernement Indien. Le succès des essais de Phase-III en cours par la preuve d’une réversibilité chez des hommes volontaires apportera au Monde une méthode de contraception développée localement.

## Family planning: the way forward

Family planning is crucial for the achievement of sustainable development goals and subsequent efforts need to be made to improve access and strengthen quality of family planning services. Research shows that adequate attention to family planning in countries with high birth rates can not only reduce poverty and hunger but can also avert maternal and childhood deaths [1].

In 2012, the global community at the London Summit on Family Planning formulated a global partnership ‘Family Planning 2020’ (FP2020), with an aim to add 120 million women and girls to the category having access to effective and safe family planning methods and services by the year 2020. Towards achieving the FP2020 goal national governments, civil societies and the private sectors joined hands to address the barriers that affect access and use of contraceptives. According to FP2020, modern methods of contraception can prevent a large number of unintended pregnancies, unsafe abortions and maternal deaths [2]. In the FP2020 focus countries modern contraceptive prevalence rate was estimated to be 45.7% in 2017 with 21.6% unmet need for modern contraceptive methods. Usage of modern contraceptive methods by women globally, increased by 28.8 million between 2012 and 2017. Modern contraceptives are the most relied method in Western Europe with 95.5% population using condoms, hormonal contraceptives or sterilization and only 4.5% users rely on traditional methods. In Eastern and Central Europe users of modern methods of contraception is 77.5% with rest of the population (22.5%) still following traditional methods of contraception [3]. India’s contraception prevalence rate among all women was 39.2 in the year 2012, 39.57 in 2017 and is predicted to rise to 40.87 by the year 2020 [2]. About three-fourths of these were using female sterilization, which is by far the most prevalent birth-control method in India. However, role of male partner in family planning has been highly limited, specifically in developing nations like India.

Traditional methods of male contraception have long included periodic abstinence, non-vaginal ejaculation, condoms and vasectomy [4]. The lack of modern methods of contraception for men does not, however, explain the low prevalence of male sterilization, given that vasectomy is more effective, less expensive to perform and has fewer complications than female sterilization [1, 5]. However, for men to share more equally the burdens as well as the benefits of family planning, more effective reversible male contraceptive methods need to be available. The resultant diminished male role may have inadvertently undermined the many societal efforts at birth control. Many men, young and old, still perceive contraception as primarily a woman’s responsibility, for after all, she suffers most directly from contraceptive failure; this attitude is unfortunate [6]. Since decisions about pregnancy affect both partners, both should share the contraceptive burden equitably. Limited choices and access to methods, attitudes of men towards family planning, perceived fear of side-effects, poor quality of available services, cultural or religious oppositions and gender-based barriers are some of the reasons for lesser participation of men in family planning.

## Methods of contraception for male with limitations

In 1950s and 1960s males were overlooked by family planners even after being an integral part of the family unit [7]. Drug companies were reluctant to invest in developing contraceptives for male consumers. There were various misconceptions and misbelieves regarding side-effects like loss of libido and so called “manhood”. Further, there were unproven assumptions regarding male attitudes in sharing responsibility of family planning [8]. Development of male contraceptive thus lagged behind due to both societal and technological stereotypes. Considering the dismal past of male contraceptive research, in 1960 R. J. Ericsson, an early pioneer in male reproductive research, quoted it as “almost an illegitimate specialty within reproductive biology” [9]. Present available contraceptive methods for male have been listed below with their advantages and disadvantages (Table 1).

- **Male condoms**: Condoms are made from very thin latex (rubber), polyisoprene or polyurethane. Condoms are being practiced by people worldwide for contraception and prevention of sexually transmitted diseases as they are cheap and easily available [11]. They are associated with infidelity, reduce the spontaneity and sensitivity of sexuality, present problem of storage and disposal and have high failure rate (3–15%). Condom failure due to condom breakage, slippage, incorrect use and latex allergies also occur [12, 26].
- **Coitus interruptus (Withdrawal):** Coitus interruptus is the practice of ending sexual intercourse before ejaculation. The main risk for coitus interruptus is related to perform correctly or in a timely manner. Disadvantages of this method include the fact that it requires high motivation and is highly frustrating to some couples. Another disadvantage is that any sperm deposited before withdrawal, or left on the vulva wall during withdrawal, could reach the cervix. These factors account for the high failure rate of coitus interruptus [10].
- **Hormonal approaches:** The hormonal approach is based on the reversible suppression of gonadotropins leading to reversible suppression of the spermatogenetic process. Over the last decades studies have been performed to evaluate the level of acceptability of possible hormonal methods for male contraception. Medroxyprogesterone acetate is a hormonal medication of the progestin type that is shown to prevent spermatogenesis in combination with the topical application of testosterone gel [13]. Testosterone enanthate in clinical trials showed good efficacy with few drawbacks [14]. Most of the hormonal approaches have reached to clinical trials, but none of them has been approved for acceptability in public use. Major drawbacks in use of male hormonal contraceptive regimens are side effects like proatherogenic or antiatherogenic action, association with insulin resistance, hematopoetic action, etc. World Health Organization (WHO) conducted trials with men who receive twice weekly injections of testosterone. As noted earlier, this has the effect of suppressing sperm production by decreasing levels of gonadotropin-releasing hormone (GnRH), follicle-stimulating hormone (FSH) and luteinizing hormone (LH). Good results were obtained, but they found that the frequent injections posed a commercial and psychological barrier. More troubling, high levels of circulating testosterone led to increased irritability, acne and reduced levels of good cholesterol in many test subjects [15]. A recent review on male contraception highlighted that delay in development of male hormonal contraception is multifactorial. However, willingness to use a new male method is driving new clinical trials closer than ever to bringing viable products to market [16]. Thus, no hormonal regimes have yet been approved for contraceptive use and focus has been shifted from hormonal to non-hormonal strategies.
- **Immunocontraceptives:** Immunocontraception involves the administration of vaccine that induces an adaptive immune response which causes an animal to become infertile. The method promises for high target specificity, long term action but not permanent, relatively inexpensive, lack of endocrine or metabolic side effects, easy to use without surgical intervention. The immune system is employed as a contraceptive by targeting sperm- or egg-specific proteins, or even gonadotropins because antisperm antibodies can play a role in infertility. Few examples of target proteins for immunocontraception are SPAM1, MDC,
  SP-10, FA-1, SP-17, NZ-1, NZ-2, LDH-C, SAGA-1, hESP, rSMP-B, SAMP-32, 80 kDa HSA, BS-17, EP-20, DE Protein, SFP2, AKAP, TSA-1, YLP-12 and Izumo [17, 18]. The development of immuno-contraceptives is still at the research stage. The future of contraceptive vaccines holds great promise in terms of comfort, price, efficacy, complications, and possibly non-selective action in animal populations as well as in humans.
- **Non-injectable plugs:** Silicone plugs, called the Shug is composed of two silicone plugs with nylon tails to help anchor the plugs to the vas. Double plugs could be more reliable than the single one. The Shug can be inserted into the vas by the no-scalpel method and removed by minor surgery. In monkeys, fertility was returned after seven months of Shug use [19]. Clinical trials reported in men with 97% reduction in sperm motility. The Shug has several advantages: the size of the plug could be controlled according to the size of the vas deferens, thus avoiding the possible rupture of the vas; the anchoring mechanism can prevent the migration of the plug along the length of the vas. The preformed plug also avoided the possibility of entry of toxic substances during the hardening processes as in the case of injectable silicone rubber [20].
- **Vasectomy/Male sterilization:** Vasectomy is a safe and effective mode of permanent male contraception used by 42–60 million men worldwide. Vasectomy is safe with no mortality, effective, simple, convenient, requires only 10–15 min and inexpensive compared with female sterilization, which relatively costly and risky. Minor side effects are bruising, scrotal swelling, acute pain, hemorrhage, haematoma and surgical infection. But there are many reasons for its low acceptance. There is a possibility of prostate cancer after 20 years of vasectomy, due to enhanced dihydrotestosterone levels [27]. Issue of reversal on desire which requires skilled microsurgery and is less assured due to sperm antibodies development. It provides no protection against STD, and reversal is expensive with only partial success [22, 28].

**Table 1:** Methods of male contraception

Method	Advantages	Disadvantages	References
Abstinence	No side effects. No cost.	Difficult to abstain for long duration.	[4]
Withdrawal	No Cost.	High risk of pregnancy if not withdrawn at time. Pregnancy may occur by pre-ejaculate.	[10]
Male condoms	Easy availability. Helps in prevention of STIs.	Decrease spontaneity. May break during use. High failure rate.	[11, 12]
Hormonal approaches	Non-surgical procedure.	Lack of uniform efficacy, Complex formulations, Impractical systemic delivery system, Poor availability, High cost	[13–16]
Immuno-contraceptives	Target specific effect. Long-term efficacy. No surgical interventions.	Still under research phase.	[17, 18]
Non-injectable Plugs	No-scalpel method. Size available according to vas, thus avoids vas rupture.	Lower efficacy. Delayed azoospermia Reversal – less assured	[19, 20]
Vasectomy	Safe and effective. Risk involved in surgical intervention.	Microsurgical skills required. Antisperm antibody development. Reversal is expensive and partially successful.	[5, 21]
Non-Scalpel Vasectomy	No surgical procedure. Easy technique. High efficiency.	Reversal is expensive and partially successful.	[22, 23]
RISUG^®^	Easy approach. Single intervention. Early contraception Minimal systemic interference. No undue side effects. Better scope for reversal.	No protection against Sexually Transmitted Diseases (STDs).	[24, 25]

### Conventional vasectomy

**Fig. 1:**
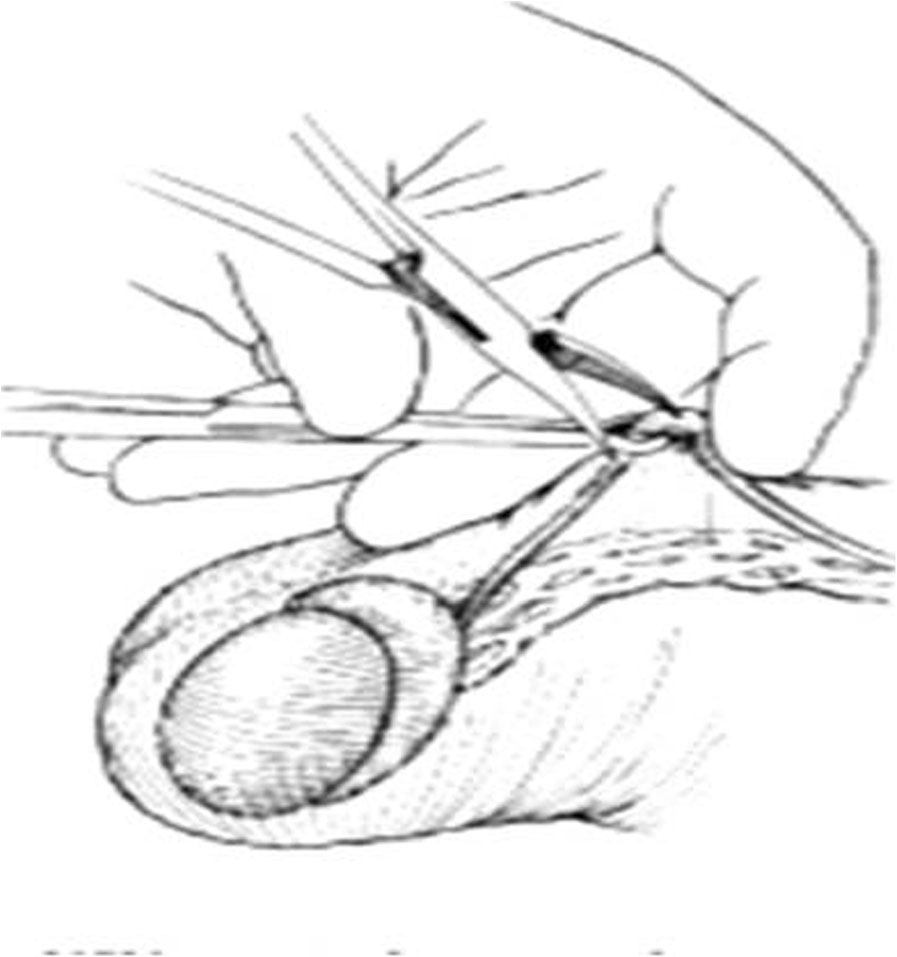
Non-scalpel vasectomy approach. The No Scalpel Ring Clamp isolates and secures the vas deferens without penetrating the skin. The No Scalpel Dissecting Forceps pierces the scrotal sac to expose the vas deferens. The vas deferens is lifted out of the scrotum with the No Scalpel Dissecting Forceps and occluded

Involves bilateral scrotal incisions through which the vas deferens is mobilized and transacted. This approach is effective but difficult to be reversed [29]. **No-scalpel vasectomy (NSV)** uses a unique puncture technique that reduces trauma to the scrotum and vas deferens (Fig. 1). The urologist uses a special clamp to puncture the scrotal skin, retrieve the vas and separate it from the surrounding structures in the scrotal sac without cutting the nerves or blood vessels near the scrotum. NSV is associated with no incision, no stitches, faster procedure, faster recovery, less chance of bleeding, less discomfort and high efficiency, which have helped the technique to increase the acceptability of male sterilization in many parts of the world [22, 23].

Vasovasostomy is a form of microsurgery first performed by the Australian Surgeon, Dr. Earl Owen in 1971 [5]. Pregnancy occurs in approximately 50% of couples after vasovasostomy and 30% of couples after vasoepididymostomy within 1 year of vasectomy reversal. Approximately 50 - 80% of vasectomies men develop antisperm antibodies [30]. A scar tissue develops at the site of reversal causing a blockage in 5–10% of vasovasostomies [31]. The vasectomy reversal will probably fail if an epididymal blowout has occurred at the time of vasectomy reversal surgery. The epididymis is adversely affected by elevated pressure due to long time vas deferens blockage that results in poor sperm motility [32]. NSV procedure requires surgical skills, handling of special instruments and manual skills. There are also physiological effects of vasectomy on male reproductive system that makes vasectomy a potentially permanent method of contraception [21].

Currently, there are several promising traditional, herbal, hormonal, non-hormonal, immune based and vas based contraceptives at various stages of research and development. There is clearly a desire and need for more contraceptive options [33]. Couples desire more choices for fertility control, and unplanned pregnancies continue to occur at alarming rates. Through further research, advocacy and support, male contraceptives are likely to become a valuable addition to the current choices of family planning. The shortcomings of currently few available male contraceptive methods are a major barrier to the involvement of men in family planning. Current research into male contraceptives will potentially increase the equitability of family planning between males and females.

### Birth of RISUG^®^ (reversible inhibition of sperm under guidance)

The limitations of available male methods in controlling birth rate raise the idea to develop a new method which overcome the constraints and provide an ideal method to use. Prof. Sujoy K. Guha, School of Medical Science and Technology, Indian Institute of Technology, Kharagpur, India known for his innovative techniques worked on developing a method for stimulating the flow of blood through the human body. Later, his idea was utilized by ship manufacturers to design a pump known today as magneto-hydrodynamic propulsion units. In the 1970s while investigating some cost effective techniques to purify rural water systems, he discovered that when pipes were coated with a common polymer called styrene maleic anhydride (SMA), it could kill bacteria present in the water supply. In concert with Government of India, Prof. Guha worried regarding rapidly growing population of the country and suggested use of SMA to be developed as a male contraceptive [34, 35].

The proposed design was modified to work safely inside male genitalia and then considering, vas deferens similar to a water pipe and sperm travelling through the narrow tubes analogous to microbes, reproductive tract of male rats were injected with SMA. Positive results, indicated by sterility in rats, were observed and published in 1979 [34]. Later on, the procedure was further refined and also tested in rhesus and langur monkeys.

### Journey begins as male contraceptive

In 1979, Prof. Guha proposed a radically new technique of male contraception, styrene maleic anhydride (SMA), a co-polymer dissolved in dimethyl sulphoxide (DMSO) was injected into vas deferens in rats. RISUG**®** (Reversible Inhibition of Sperm Under Guidance), a co-polymer of SMA dissolved in DMSO, was developed as a new perspective in non-hormonal male contraception methods [24]. RISUG**®** was formulated as an occlusive polymer which was claimed to sterilize subjects by single injection and reversed at any time following vas occlusion. Within 72 h of injection, RISUG**®** forms electrically charged precipitates in the lumen and further layers the lumen wall and inner walls of vas deferens. Precipitates are dominated with positive charge creating an acidic environment. Passing through the RISUG^®^ injected vas deferens, sperms suffer ionic and pH stress, causing acrosomal damage, rendering them unable to fertilize oocytes. Afterwards it was demonstrated that SMA polymer injected into vas deferens of rhesus monkey could occlude the vas deferens lumen and also inhibits the fertilizing ability of spermatozoa by virtue of the pH lowering effect. Preclinical toxicity evaluated in rodents (Charles Foster rats) showed safety of the compound [36]. The polymer was proposed to be injected into the vas deferens through the non scalpel procedure thus avoiding surgery in the initial sterilization procedure. After being introduced in 1980 successful pre-clinical efficacy and safety studies on various species of animals including primates, RISUG**®** has also been tested successfully in number of human volunteers during Phase-I, Phase-II and Phase-III clinical trials. Presently the drug is under extended Phase-III clinical trials at various centers in India.

### Composition

RISUG**®** is synthesized by dissolving 60 mg SMA in 120 μL DMSO. The DMSO is strongly alkaline and hygroscopic, minimal concentrations reported with no cytotoxicity. DMSO was chosen as solvent vehicle as it helps the penetration of polymer into the folds of inner wall of the vas deferens and its retention [24]. A part of SMA is converted into styrene maleic acid, which neutralizes the alkaline pH of DMSO. This action reduces the reactivity of DMSO. However, because of the sulphur moiety, DMSO is highly reactive. When SMA is mixed with this particular form of DMSO, the sulphur moiety of DMSO interacts with the etheric oxygen (−O-) of maleic anhydride moiety of SMA thereby leading to the formation of an intermediate unstable complex of SMA and DMSO. The carbonyl oxygen of SMA being resonance stabilized is not affected.

### Mode of action

**Fig. 2:**
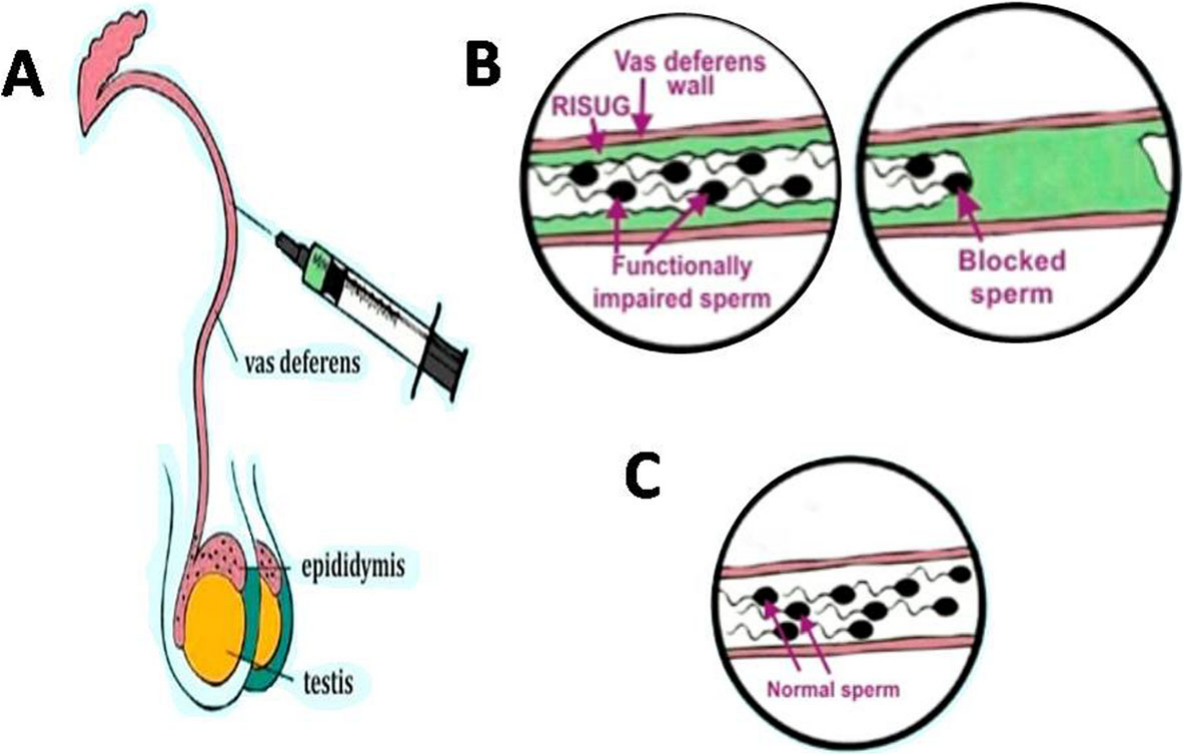
RISUG: mode of action. (**A**) Vas is exposed from inguinal region and RISUG is injected in both vas deferens towards distal region by a micro-syringe. (**B**) RISUG coats the wall of the vas deferens blocking sperm movement. (**C**) Complete reversal obtained after DMSO/NAHCO_3_ is injected bilaterally, flushing component of RISUG [37]

The complex of SMA and DMSO was suggested to act through vas occlusion, pH lowering and charge disturbance effect [25]. When RISUG**®** is injected into the vas deferens, it comes under the influence of the proteins in the spermatic fluid of the vas deferens. The polar amino acids react with the SMA - DMSO complex. Due to the formation of SMA - DMSO complex, there is a chemical instability which enables the polar amino acids to detach DMSO from SMA, while retaining the broken bond and polyelectrolyte nature of SMA. Thus, the COOH of maleic anhydride exists as COO^−^ and H^+^. The reactions with the proteins take 48 h to complete. During this period, the DMSO helps in the entry of SMA into the folds of the vas deferens inner wall which promotes anchorage and retention of the contraceptive (Fig. 2). When the reaction is complete all the DMSO is detached and gets absorbed into the surrounding tissue and the blood stream for ultimate secretion [24]. The place of DMSO is then taken over by the proteins of the spermatic fluid with the polar amino acids of the proteins linked to the SMA and sustaining the polyelectrolyte nature induced into the SMA. The negative charge of COO^−^ ions and the positive charge of H^+^ ions are maintained in a bound state. The proteins form a layer around SMA. An electrical charge double layer formation occurs with the proteins covering the SMA. The amino acids of proteins are zwitter ions having both positive and negative charged groups. In SMA, COO^−^ ions are structurally larger than the H^+^ ions, but are less active. The more active H^+^ ions tied up with the negative charged groups of amino acids, thus rendering them less effective in giving an external charge. Hence, the positive charges of the amino acids are left to give an external influence.

The protein - SMA complex has a positive charged surface which can influence the sperms. Also the protein layer over the SMA gives a protection to SMA from dissolution. This phenomenon gives the long-term contraception in the vas deferens. The hydrolyzed RISUG**®** in the vas deferens is claimed to have a pH of 4.0–4.5 which is likely to lower the motility, but would it completely immotile the sperms is an important question. The sperm damages due to RISUG**®** are very much similar to that of the damages caused by oxidative stress. The generation of excess of intracellular or extracellular reactive oxygen species (ROS) such as, O_2_^−^, H_2_O_2_, ROO^•^, OH^•^ are associated with many cell damages, including morphological defects, DNA fragmentation, lipid peroxidation, decrease in acrosome reaction and fusiogenic ability and impaired fertilization [38–41]. The concept of implantation of the SMA in the rat vas deferens have been confirmed by transmission electron microscopic (TEM) examination and fluorescence microscopy of vas fluid and prostate tissue [42]. In extended Phase III clinical trial, 60 mg styrene maleic anhydride dissolved in 120 μL of DMSO (1: 2) induced azoospermia in 84% of the subjects with presence of occasional abnormal sperm along with low neutral α-glucosidase activity was indicating ‘partial’ and not ‘complete’ vas occlusion. However, no further study was published to support this statement [43].

### Preclinical trials

Various animal models have been used for attaining contraception with RISUG**®**, through vas occlusion, before initiation of clinical trials, for the safety evaluation with contraceptive effects.

- **Rat:** In an early study, co-polymer of styrene and maleic anhydride was dissolved in DMSO and injected into the vas deferens of rats. Histological observations indicated that the polymer was retained in the vas deferens and the morphological changes detected were confined to the mucosa. When the polymer was removed by flushing DMSO, the mucosal structure became normal within 2 weeks [44]. Later, further studies were carried out in rats related to its reversibility aspects.
- **Rabbit:** For the first time, SMA was evaluated in male rabbits as a contraceptive by Sethi et al. and the results showed no teratogenic potential at the doses of 1.25 mg, 2.5 mg and 5.0 mg used in the experiment [45].
- **Rhesus monkey:** SMA, was injected in to the vas deferens of male rhesus monkeys for safety evaluation at the dose of 100 mg (contraceptive dose, CD), 250 mg (CD × 2.5) and 500 mg (CD × 5.0). The observed behavioural, haematological, biochemical and histopathological parameters in treated monkeys were comparable to controls. The results suggested the polymer SMA to be safe up to 5 times CD in monkeys [46]. Similarly, another study in rhesus monkey showed that the polymer has the dual feature that it can occlude the vas deferens lumen and also can inhibit the fertilising ability of spermatozoa by virtue of the pH lowering effect. Matings with females were carried out when the lumen was completely occluded giving azoospermia as well as with partial block and spermatozoa present in the semen. All matings were infertile. Data up to 1 year were presented and indicated that the contraceptive effects last for a considerably long period [38]. Later, Guha et al. presented alterations in sperm plasma membrane, mitochondria as well as in the sperm structural components through histological data of monkey, providing a means of causing changes in the sperm that inhibit the fertilizing ability. Therefore, achieving non-obstructive vas-based contraception, without genotoxic or teratogenic effects caused by infertile sperm passing into the semen, is feasible [47].
- **Langur monkey:** The findings were also presented in langur monkeys with the changes in the physical characteristics of semen and ultrastructure of the spermatozoa after vas occlusion with SMA. Scanning electron microscopy (SEM) revealed severe coiling of tail, rupture of acrosomal envelope, and bent midpiece associated with damaged mitochondrial sheath. Observations by transmission electron microscopy (TEM) revealed vacuolization in the nucleus, membrane damage in the acrosome, loss of segmented columns, and numeric aberrations in the centriole of the neck, as well as degeneration of mitochondrial sheath and axoneme in the midpiece, and absence of outer plasma membrane in the midpiece and tail. The results indicated that the necrospermic status of the spermatozoa during initial ejaculations may offer instant sterility after vas occlusion with SMA [48]. After that, routine hematology, clinical chemistry, the serum testosterone and sperm antibody titers were studied that remained unchanged from their pretreatment values until 540 days of vas occlusion. Histology of testes revealed continued spermatogenesis throughout the study period. The results suggested focal degeneration of seminiferous epithelium in the central portion of the testis following long-term vas occlusion with SMA [49].

### Clinical journey

After fertility control investigations, toxicological studies and further successful safety evaluation on albino rats and rhesus monkeys the Indian Council of Medical Research (ICMR) and Drugs Controller General of India (DCGI) permitted to conduct Clinical Trials in 1989 (Table 2). Study was planned to assess the contraceptive effectiveness and safety of the intra-vas deferens injections of complex comprised of SMA in a solvent vehicle of DMSO.

- **Phase-I of the clinical journey**: Phase-I clinical trial was initiated at a few centers with 38 healthy adult male volunteers with normal reproductive system [50]. Female partners of all the volunteers enrolled in the Phase-I clinical trials had already undergone tubal occlusion, thus the efficacy was obtained as indirect evidence in terms of semenological studies. The Phase I clinical trial was focused at confirming the safety and side effects of the drug preparation evaluated on the basis of clinical parameters, as the drug was being used for the first time in medical studies. After complete medical examination, with local anesthesia, a small incision of about 7 mm length in the scrotal skin to the left of the midline and at a level 15–20 mm above the upper pole of testis was made. In the distal direction while maintaining proximal compression, 5 mg to 140 mg doses of the polymeric drug of SMA was injected into the vas deferens using a 23-gauge needle. After injection clinical assessment and semenology was periodically performed for more than 2 years. Drug with 60–140 mg SMA was found to be effective showing azoospermia during 20–389 days post injection. Most effective outcomes were observed with 70 mg SMA dose which showed azoospermia in nearly 3 weeks and the subjects stayed azoospermic for 292 days. The Phase-I clinical trial of RISUG® with more than 2 years of follow-up study demonstrated that the procedure does not lead to any clinical complications in the urogenital system and other parts of the body.
- **Phase-II of the clinical journey**: Phase II clinical trials with RISUG**®** injection were initiated to assess efficacy of intravasal injection based on azoospermia and no pregnancy in the female partner that have a normal reproductive profile, had not undergone sterilization and have not being using any other conventional contraceptive. Under the Phase-II study, 12 healthy adult male volunteers were injected with 60 mg of SMA. All the subjects underwent pre- and post-injection clinical examination that included sperm count, motility and morphology assessment. Results of the Phase-II studies showed injection with 60 mg of SMA can induce azoospermia immediately and was observed for more than 12 months. Azoospermia was observed in all the subjects with no side effects [51, 54]. Female partners of all the subjects retained good health throughout the study and no pregnancies were reported during the period of study [51].
- **A parallel journey:** In Phase-II, 60 mg of SMA resulted in an immediate contraceptive effect, parallel a 2 year clinical efficacy trial was performed with variable doses of RISUG^®^ [54]. The study included 20 subjects who were injected with 40, 50, 60, 65 and 70 mg of SMA and were monitored for the maximum of 1407 days. Results of the study suggested dosages ranging from 40 to 70 mg of SMA were effective in giving more than 2 years of contraception regardless of azoospermic or non-azoospermic stage of the subjects. One subject amongst the 20 subjects under study had a normal child after 145 days of injection, due to slippage during injection. All subjects maintained good health during the course of vas occlusion with RISUG^®^, indicating efficacy and safety of the drug.
- **Phase-III of the clinical journey**: Long term follow up of human volunteers with RISUG^®^ during Phase-II clinical trials showed the method to be effective and safe. However the study involved only a limited number of volunteers, thus Phase-III clinical trials were designed to evaluate safety and effectiveness of intravasal RISUG^®^ injections in a larger sample.

**Table 2:** Clinical journey of RISUG^®^

	No. of Subjects	Dose regimen	Sperm count (million/ml)	Remarks	References
Phase I	38	5 mg to 140 mg	For 60–140 mg dose azoospermia was reported during 20–389 days post injection	Phase I clinical trial showed that the injection of DMSO and DMSO-SMA mixture into the lumen of the vas deferens is a safe procedure with no long-term adverse effects.	[50]
Phase II	12	60 mg	All subjects were azoospermic within 5–243 days	The results of Phase II clinical trials reconfirm the safety and show that for a period of at least one year, the treatment leads to azoospermia in the male and gives pregnancy protection.	[51]
Phase III	315	60 mg	After 2.5 months 92.6% subjects and after 6 months 96.7% subjects showed azoospermia post RISUG® injection.	Contraceptive efficacy was found to be 99.02% with 0.3% method failure and 0.98% overall failure in the drug efficacy.	[52, 53]

In 2003, a brief study was performed wherein 25 healthy adult male volunteers were injected 60 mg SMA dissolved in 120 μL of DMSO [43]. Based on assessment of levels of neutral α-glucosidase, the biochemical marker for epididymis, acid phosphatase activity and fructose levels in the seminal plasma, RISUG^®^ was shown to be effective as a partially occluding agent in the vas deferens. Semen and biochemical analyses were done for a period of 6 months post-injection and the results showed predominantly showing immotile and abnormal spermatozoa in all subjects after injection of RISUG^®^.

Phase-III clinical trials with RISUG^®^ were initiated by ICMR at four different centers in the country with 64 healthy adult male volunteers [55]. All the subjects were injected with 60 mg SMA dissolved in 120 μL of DMSO and were followed up for efficacy and safety. Post injection, all the male volunteers and their female partners underwent clinical evaluation after 3, 7 and 21 days. Further, clinical and laboratory examinations that included monitoring for infections, pyrexia, pain and/or swelling in scrotum, liver and kidney function tests, blood and urine examinations, ultra-sonography of scrotum/lower abdomen and vital organs, etc. were performed and noted for all the subjects at 1.5 months, 2.5 months, 4 months, 5 months, 6 months and then after every 6 month interval till 5 years post RISUG^®^ injection. ICMR reported no side effects of the drug with all subjects maintaining high clinical efficacy. 92.6% of subjects were reported to achieve azoospermia at 2.5 months post injection and in 96.7% of the subjects azoospermia was reported at 6 months after RISUG^®^ injection [52]. In 2018, a total of 315 subjects enrolled at 5 different centers in the country were reported to show no adverse side-effects of the drug with overall contraceptive efficacy of 99.02%. Few subjects were reported to be lost in follow up due to personal reasons, 0.3% method failure and 0.98% overall failure in efficacy of the drug has been observed [53]. A multi-centric limited Phase III clinical trial of RISUG^®^ reported no pregnancy among the subjects that received complete dose of RISUG^®^ and indicated it is an effective and safe male contraceptive with majority of the individuals under study achieving either oligozoospermia or azoospermia within 2 months after injection [56].

## Advantages of RISUG^®^ over other methods of male contraception

RISUG^®^ creates a physical and chemical barrier preventing sperm from reaching the oocyte. The polymer is injected into the vas deferens through the non scalpel technique, thus avoiding surgery in the initial sterilization procedure. There are few major advantages of RISUG^®^ as mentioned below, that has made it a potential male contraceptive.

- **Early azoospermia:** Contraception is an all or none game, a single sperm is sufficient for fertilization resulting in an unplanned pregnancy. Three most commonly used methods of male contraception that have been in use for hundreds of years present a very high first-year failure rates (periodic abstinence - 20%, withdrawal - 19%, condoms - 3-14%). Vasectomy is a surgical method of male sterilization considered to be highly effective and permanent form of contraception. However, absence of sperms in ejaculates is mostly observed at-least 12 weeks after the procedure. Clinical trials with RISUG^®^ demonstrate promising results showing azoospermia in subjects as early as 4 weeks after the injection that is sustained over years. A few sperms that are observed in ejaculates after RISUG^®^ were found to be functionally inactive [25].
- **Reversibility**: In any contraceptive method a great concern is the reestablishment of fertility when required. RISUG^®^ presents an advantage over other male contraceptive methods like vasectomy with its effective and easy reversibility, as observed in different animal models. Removal of SMA co-polymer (RISUG^®^) can be induced by injecting DMSO or NaHCO_3_ that acts as partial solvent. After preclinical trials in various animal models based on blockage of vas deferens without any toxicity, the studies have been moved toward its reversibility aspect without affecting cellular integrity. Despite the promising results of reversibility in animal models, the reversibility studies have not yet been carried out in humans.

i. **Rat:** The SMA polymer was removed by flushing dimethyl sulphoxide in vas occluded rats and observed that the mucosal structure of vas deferens became normal [44, 57]. Subsequently, the functional success and safety of vas occlusion reversal by DMSO was reported in rat model along with teratogenicity studies [58]. After that, sodium bicarbonate (10%), pH 8.9, was used to flush the polymeric material from the vas deferens lumen in rats. Histological observations of vas deferens indicated potential role of NaHCO_3_ in reversal of vas deferens blockage [59]. The reversal with NaHCO_3_ in rats resulted into an early resumption of fertility when compared with DMSO and the procedure found to be successful, feasible and safe up to F_1_ generation [60]. It is also concluded that vas occlusion with RISUG^®^ at the contraceptive dose regimen is not associated with genotoxicity in leukocytes or the testis of pre- and post-reversal rats [61]. The study using both DMSO and NaHCO_3_ for reversal of RISUG^®^-induced contraception was successful without any toxicity at the cellular levels [62].
ii. **Rabbit:** The results of reversal studies in rabbit suggested that DMSO and NaHCO_3_ were feasible, with normal progeny, following short- and long-term contraception. The safety evaluation following vas occlusion with RISUG^®^ and its reversal using genotoxicity tests and apoptotic marker assays concluded that it has not been correlated with any toxicity [63, 64].
iii. **Langur monkey:** Non-invasive reversal approaches (palpation, percutaneous electrical stimulation of the vas deferens, forced vibratory movement, suprapubic percussion and per rectal digital massage of the vas deferens) have been applied in monkeys. The results suggested that non-invasive reversal was feasible even after long-term vas occlusion with SMA and is safe without adverse side effects [65–67]. Ultrastructural changes in the vas deferens of langur monkeys after 150 days of vas occlusion with styrene maleic anhydride (SMA) and after 150 days of non-invasive reversal were also reported. The results suggested that the exfoliation of the epithelium due to vas occlusion by SMA regains normalcy after 150 days of non invasive reversal [68, 69]. Degeneration of seminiferous epithelium was evident in some of the tubules and following 420 days of vas occlusion, the central portion of the testis showed regressed seminiferous tubules depicting various shapes and devoid of germ cells, which continued until 540 days of vas occlusion [69].
iv. **Other added advantages**: In the field of contraception, RISUG^®^ has several advantages such as effectiveness, no interruption before the sexual act, cost factor, outpatient procedures means patients can leave the hospital immediately after an injection and resume their normal sex lives within a week, duration of effect that for at least 10 years no side effects with greater reversibility. Apart from male contraception, RISUG^®^ shows antibacterial effect on *Escherichia coli* illustrated through a SEM, TEM and AFM based study. RISUG^®^ based on its composition i.e. SMA, was hypothesised to demonstrate antimicrobial activity against various microorganisms like *Candida albicans, Pseudomonas auroginosa, Staphylococcus aureus, Escherichia coli,* etc. [70]. It has been also suggested that viruses could be more sensitive towards antimicrobial action of RISUG^®^ than bacteria and based on this assumption RISUG^®^ has been presented as a potential candidate for developing antiretroviral drug/ male vas deferens implant for HIV free semen [70, 71]. RISUG® was thus taken as a potential antiretroviral drug, still study needs further confirmation and mechanism needs to be elucidated.

### Female contraception with RISUG^®^

A recent study was initiated to examine the tissue specific reaction and the histo-architecture of the female tract that receive the polymer implant. The above finding indicates that the drug is compatible within the fallopian tube and therefore needs to be explored further for its contraceptive potential in females [72]. The contraceptive efficacy of intratubular injection of RISUG**®** and its reversal assessed in female rats was found to be safe without any untoward side-effects [73].

### Why the drug is still not in market after 3–4 decades of research?

Towards answering this one must understand that regulatory measures take time and these requirements help protect people from potentially harmful products. Looking for an alternative, effective and reversible male contraceptive, hormonal methods of male contraception were developed, but none could reach the markets due to undue side effects, lack of uniformity in results and also need for long-term administration. Preclinical and clinical journey of RISUG**®** demonstrates high efficacy and safety of the drug. With regard to reversibility, safety and efficacy trials have been performed only on animal models. Before putting RISUG**®** into market, its reversibility needs to be clinically verified. Another major concern inhibiting the progress of RISUG**®** is lack of interest from pharmaceutical industries. In 2000, a survey found 83% of men from various countries are willing to accept male contraceptive. Despite, pharmaceutical companies are reluctant to pursue the idea to avoid losing the thriving global markets for female contraceptives and condom that value to billions each year. Initially, RISUG**®** attracted some interest from pharmaceutical companies. However, considering it as an inexpensive one time procedure manufactures retracted. Taking in to account the ever increasing population of countries like India, there is a demand for family planning, thus RISUG**®** caught attention of the Government. Apart from scientific and monetary matters, major hindrance that stands in the way of this revolutionary male contraceptive is men itself. In the male dominating society it has always being tough for men accepting the responsibility of family planning. Today the world communities are evolving and there is increased focus on involvement of men from supporting and understanding female partner’s reproductive health to engaging men as contraceptive users. Various studies are coming up engaging men as potential clients of family planning and surveys indicate about young adults being more willing to use male contraceptive methods [74, 75]. The perspective also varies by country and demographical backgrounds; a wider acceptability has been reported amongst men with advanced educational background and stable employment [76]. Nevertheless, scientists are pushing ahead and the momentum and buzz in the field is reflecting fresh optimism.

## Conclusion- present scenario and future perspectives

The RISUG® has surely created a new concept of contraception with great feasibility and long lasting sterility. After being introduced in 1980 successful pre-clinical efficacy and safety studies on various species of animals including primates, RISUG**®** has also been tested successfully in number of human volunteers during Phase-I, Phase-II and Phase-III clinical trials. Presently the drug is under extended Phase-III clinical trials at various centers in India waiting for approval from DCGI for mass production. Although, many leads have been taken towards making of an effective male contraceptive, many of these failed, many of these succeeded at first and then failed, many are still struggling for recognition, RISUG**®** on the other hand, provides a hope which has a slow pace and drawbacks, but it is in a right direction.

## Data Availability

Not applicable.

## Abbreviations

AFM: Atomic Force Microscopy
CD: Contraceptive Dose
DCGI: Drugs Controller General of India
DMSO: Dimethyl Sulphoxide
FAMS: Fellow of National Academy of Medical Sciences (India)
FEMSI: Fellow of Electron Microscopy Society of India
FIAES: Fellow of Indian Academy of Environmental Sciences
FNASc: Fellow of National Academy of Sciences, India
FP2020: Family Planning 2020
FSH: Follicle-Stimulating Hormone
GnRH: Gonadotropin-Releasing Hormone
HIV: Human Immunodeficiency Virus
ICMR: Indian Council of Medical Research
LH: Luteinizing Hormone
NASI: National Academy of Sciences, India
NSV: No Scalpel Vasectomy
RISUG**®**: Reversible Inhibition of Sperm Under Guidance
SEM: Scanning Electron Microscope
SMA: Styrene Maleic Anhydride
STDs: Sexually Transmitted Diseases
STIs: Sexually Transmitted Infections
TEM: Transmission Electron Microscopy
WHO: World Health Organization
